# The effect of statins on the survival of patients with amyotrophic lateral sclerosis: a meta-analysis

**DOI:** 10.3389/fneur.2026.1753992

**Published:** 2026-04-01

**Authors:** Hui Zhou, Ting Meng, Dan Liang, Yuanyuan Wang

**Affiliations:** Department of Pharmacy, West China Hospital, Sichuan University, Chengdu, China

**Keywords:** amyotrophic lateral sclerosis (ALS), hydroxymethylglutaryl-CoA reductase inhibitors, statins, survival, motor neuron disease

## Abstract

**Background:**

Amyotrophic lateral sclerosis (ALS) is a progressive neurodegenerative disease with limited disease-modifying therapies and a poor overall prognosis. Statins, are commonly used for dyslipidemia, and have been proposed to exert cholesterol-independent actions including anti-inflammatory and potential neuroprotective effects. Prior studies, However, existing studies offer conflicting results regarding their impact on ALS survival. This systematic review and meta-analysis aimed to evaluate the association between statin use and survival outcomes in patients with ALS.

**Methods:**

A comprehensive literature search was conducted in PubMed, Scopus, and Web of Science from inception to September 2025. Studies were included if they reported survival outcomes for statin users vs. non-users among patients with ALS. Data on hazard ratios (HRs) were extracted and pooled using fixed- or random-effects models, depending on heterogeneity. Meta-regression and sensitivity analyses were performed to explore the influence of covariates such as age and gender.

**Results:**

Six studies with 3,739 participants (889 statin users) met the inclusion criteria. The pooled analysis showed no statistical significant association between statin use and ALS survival [Log(HR) = −0.04; 95% CI: −0.18 to 0.10], with moderate heterogeneity (*I*^2^ = 24.85%).

**Conclusion:**

The pooled estimate in this meta-analysis did not show a statistically significant association between statin use and ALS survival; however, the evidence is limited by heterogeneity in statin exposure definitions and likely residual confounding in predominantly observational data. Further high-quality studies with large sample sizes are needed to determine whether specific subgroups may benefit/harm from statin therapy.

**Systematic review registration:**

https://www.crd.york.ac.uk/PROSPERO/view/CRD420251160344; PROSPERO: CRD420251160344.

## 1 Introduction

Amyotrophic lateral sclerosis (ALS) is a progressive neurological condition that impairs both upper and lower motor neurons, leading to symptoms such as muscle weakness, twitching, and eventually loss of voluntary movement and respiratory failure. It typically starts between 40 and 70 years of age with a slight predominance in males. It has a poor prognosis, with a median survival time of 2–5 years after diagnosis, with no cure available and only a few treatment options that focus primarily on symptom management and slowing disease progression (1, 2). Riluzole, edaravone, and tofersen are among the few FDA-approved treatments for ALS, though their clinical benefit in significantly altering disease progression remains limited. However, their effectiveness in modifying the disease trajectory is modest at maximum (3). The pressing need for effective therapies has prompted investigation into supplementary treatments, including repurposing existing medications to enhance survival and quality of life for patients with ALS (4).

Due to antioxidant, anti-inflammatory, and possible neuroprotective roles, statins have been proposed as potential therapeutic agents in neurodegenerative conditions such as ALS (5, 6).

Nonetheless, the clinical evidence about the effectiveness of statins in ALS remains inconclusive. Numerous observational studies and clinical trials have investigated the correlation between statin usage and ALS survival (7–9). However, the results have been inconsistent. Some research indicates that statins may offer survival advantages and slow disease progression, whilst others demonstrate minimal or negligible effects or even potential harm (10, 11). For instance, Weisskopf et al. (11) indicated that early and long-term statin treatment could have a neuroprotective effect and prolonged survival of patients with ALS, while other studies have found no survival advantage or suggested that statins might worsen muscle function due to their muscle-related side effects (10, 12). On the other hand, dyslipidemia was reported among protective factors in ALS progression, raising the question regarding the potential benefit of statins in ALS (13). Additionally, the heterogeneity of ALS, along with variations in study design, patient demographics, types of statins, dosages, and the timing of statin initiation, has further made the interpretation complicated.

Prior work suggests that lipid metabolism may be associated with ALS susceptibility, including reports linking higher LDL with increased risk of developing ALS (14). However, lipid measures within ALS cohorts have shown heterogeneous relationships with prognosis, underscoring the need to distinguish incidence from survival when interpreting statin-related evidence (15).

Given the conflicting findings in the literature, there is a critical need for a more comprehensive assessment of the potential effect of statins on ALS survival that could be addressed by a systematic review and meta-analysis. These contradictions reveal a distinct gap in the literature regarding the role of statins in ALS treatment, particularly in relation to the factors that may affect their efficacy, including the specific statin type, the initiation of the treatment, and the presence of adjunct therapies such as riluzole.

We performed a systematic review and meta-analysis to synthesize the available evidence on the effect of statins on ALS survival to address this gap in ALS research. We aimed to provide solid and robust insights into whether statins can slow the progression of ALS and whether factors like statin type, dosage, duration of use, and concurrent therapies influence the outcomes. By pooling data from cohort studies and clinical trials, this meta-analysis will help clarify whether statins represent a viable adjunctive therapy for patients with ALS and identify key variables that may affect their effectiveness. The results of this meta-analysis can ultimately contribute to the development of more effective management options and add new insights into the possible underlying mechanisms for this devastating disease.

## 2 Methods

This systematic review was planned and reported using Preferred Reporting Items for Systematic Reviews and Meta-Analyses (PRISMA) guidance. The protocol was registered in PROSPERO prior to analysis (16). The protocol of this study was registered in PROSPERO (CRD420251160344).

### 2.1 Search strategy

We conducted database searches in PubMed, Scopus, and Web of Science from inception to September 2025. The strategy combined controlled vocabulary and keywords for ALS (e.g., “amyotrophic lateral sclerosis,” “motor neuron disease”), statins (e.g., “statin,” and “HMG-CoA reductase inhibitor”) and survival (Supplementary Table S1). In addition, the search strategy used in each database is presented in Supplementary Table S2. Reference lists of included papers and relevant reviews were also screened to identify additional eligible reports.

### 2.2 Inclusion and exclusion criteria

We included original human studies that enrolled participants with ALS and reported survival or mortality stratified by statin exposure (users vs. non-users), with sufficient data to extract or estimate a hazard ratio and confidence interval. We excluded non-original publications (reviews, editorials, commentaries, preprints), case reports/series without comparative data, animal or *in-vitro* studies, conference abstracts lacking full methods/results, and studies that did not report survival outcomes by statin exposure. Only English-written peer-reviewed publications were eligible to be included. However, potentially relevant studies were used in the discussion if relevant. Eligible studies for meta-analysis, included clinical and observational studies (including cohort or case-control studies) that provided relevant data such as sample size, survival outcomes, and adjusted hazard ratios. We only extracted relevant data on survival/mortality outcomes of patients with ALS comparing statin exposure groups (statin users vs. non-users), not ALS Susceptibility/risk/incidence.

### 2.3 Study selection

After deduplication, two reviewers (HZ and TM) independently screened titles/abstracts and then full texts against prespecified criteria; disagreements were resolved by discussion and, when needed, a third reviewer (YW). The study selection process, including the number of studies identified, screened, eligible, and included in the meta-analysis, was documented in a PRISMA flow diagram.

### 2.4 Data extraction

Data were extracted from each eligible study using a standardized extraction form. The following information was collected: first author, year of publication, study design, sample size (statin users vs. non-users), age, male percentage, ethnicity, diagnostic criteria, inclusion/exclusion criteria, follow-up duration, exposure definition (statin type, dosage, timing, duration), type of statin evaluated (lipophilic vs. hydrophilic), ALS onset site (bulbar vs. limb), baseline ALS Functional Rating Scale (ALSFRS-R) score, ALS disease duration at baseline, other comorbidities, use of riluzole or other disease-modifying therapies, and adjusted covariates. When applicable, results were extracted for adjusted survival outcomes, and all values were converted to Log(HR) for the final analysis.

### 2.5 Quality assessment

We assessed study quality using the Newcastle–Ottawa Scale (NOS) for cohort designs, covering selection, comparability, and outcome ascertainment. The selection domain examines the representativeness of cohorts and the adequacy of exposure and baseline outcome measures. The comparability domain assesses the control of confounding factors, while the outcome/exposure assessment domain evaluates the validity and reliability of outcome measurements and follow-up duration. Each domain is assigned a score, with a maximum total score of 9 points, indicating study quality. Studies scoring 7–9 points are considered of good quality, 5–6 points fair, and 0–4 points poor. This quality assessment process helps ensure that only studies with minimal risk of bias are included in the meta-analysis, enhancing the reliability of the results (17). Studies were categorized by overall NOS performance, with particular attention to exposure measurement, follow-up adequacy, and confounding control relevant to ALS prognosis.

### 2.6 Data synthesis and statistical analysis

The meta-analysis was conducted using Stata 17.0 (Stata Corp., TX, USA). Log hazard ratios [Log (HRs)] and their corresponding 95% confidence interval (95% CI) were extracted as effect sizes for survival. When individual studies did not describe HRs and associated 95% CIs, they were estimated from the published Kaplan-Meier curves using a previously described method (18).

Given anticipated clinical and methodological heterogeneity across studies (differences in exposure definition, statin type/potency, patient populations, and treatment eras), we used a random-effects model as the primary meta-analytic approach. Random-effects models were fitted using restricted maximum likelihood (REML).

To assess potential publication bias, a funnel plot was generated for the outcome. Begg's and Egger's tests were also applied to statistically evaluate publication bias. A *p*-value of less than 0.05 was considered statistically significant. For sensitivity analysis, the robustness of the overall effect was examined by sequentially removing one study at a time (leave-one-out sensitivity analyses) to explore the impact of excluding studies with a high risk of bias or with small sample sizes.

Meta-regression analyses were performed for hypothesis generation to evaluate whether study-level characteristics (e.g., mean age and proportion male) explained heterogeneity. Certainty of evidence was summarized using GRADE, with downgrading for observational design, residual confounding, and inconsistency.

### 2.7 Certainty of evidence using GRADE assessment

The certainty of evidence for the primary outcome was evaluated using the Grading of Recommendations Assessment, Development and Evaluation (GRADE) approach. We assessed the body of evidence across the five GRADE domains including risk of bias, inconsistency, indirectness, imprecision, and publication bias, and assigned an overall certainty rating (high, moderate, low, or very low).

## 3 Results

**Figure 1:**
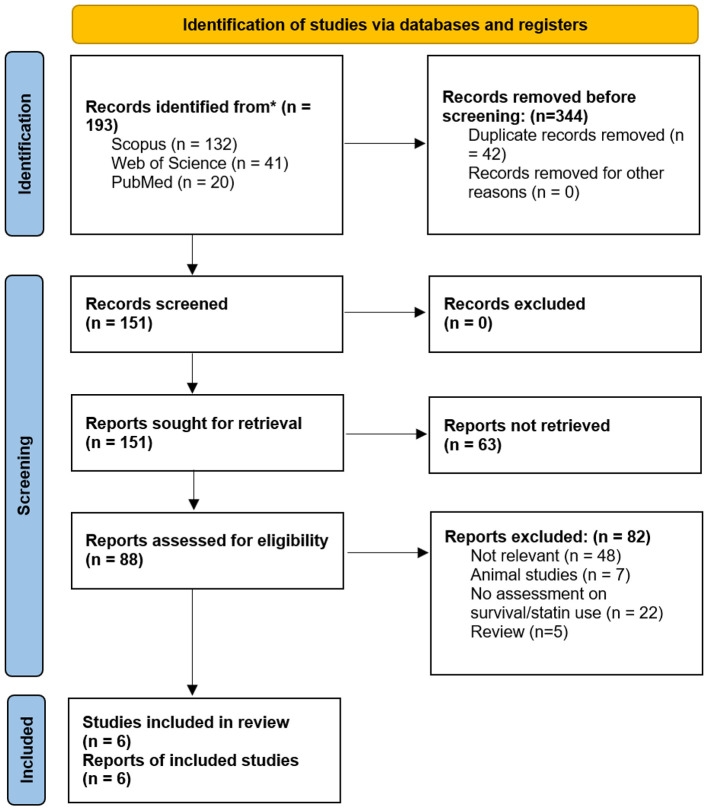
PRISMA 2020 flow diagram for this systematic review, which included searches of databases. “*” shows databases including Scopus, Web of Science, and PubMed which was searched for systematic search.

One hundred and ninety-three records were identified in our comprehensive search (PubMed: *n* = 20, Web of Science: *n* = 41, and Scopus: *n* = 132). After removing duplicate publications (*n* = 42), 151 studies were screened by title and abstract. For the next step, 88 studies were assessed for eligibility according to their corresponding full text. Reasons for exclusion at the full-text stage were documented in detail. A full list of full-text excluded studies and the corresponding exclusion reasons is provided in Supplementary Files. No additional studies were identified using Google Scholar and citation searching of relevant studies. Finally, a total of six studies met the inclusion criteria for this systematic review and meta-analysis (Figure 1). These studies included both cohort and case-control designs, and the total sample size across all studies was 3,739 participants, with 889 statin users and 2,850 non-users.

The studies were conducted in various countries, including the United States, Norway, Israel, Germany, and the Netherlands, with follow-up durations ranging from 1 to 22.3 years. The majority of studies included patients with ALS, diagnosed according to established criteria, such as the El Escorial and ICD-9 or ICD-10 criteria. The demographic characteristics of the participants varied across studies. The mean age of participants ranged from 56 to 67 years. The percentage of males ranged from 57 to 64%, and the ALS onset site was predominantly limb (ranging from 66.9 to 80.5%) or bulbar (ranging from 23.6 to 32.8%). The baseline ALS Functional Rating Scale (ALSFRS-R) score at the time of diagnosis was reported in some studies and ranged from 31.2 to 39.0. Disease duration at baseline also varied, ranging from 1.9 to 10.0 years. In terms of statin exposure, the studies varied in how statin use was defined. Statins were administered either at baseline or within a year of ALS diagnosis. The statins evaluated included simvastatin, atorvastatin, pravastatin, lovastatin, fluvastatin, cerivastatin, and rosuvastatin. Lipophilic statins, such as simvastatin and atorvastatin, were commonly used, although the exact type of statin was not specified in some studies. The duration of statin use ranged from up to 12 months to long-term use over several years, with cumulative doses varying across studies. Regarding co-interventions, riluzole use was common across most studies. The proportion of statin users receiving riluzole ranged from 74.4 to 88.9%. The use of other disease-modifying therapies or comorbidities, such as diabetes and cardiovascular diseases, was reported in some studies. Adjusted covariates included age, gender, site of ALS onset, baseline ALSFRS score, comorbidities, and use of other medications such as riluzole (Table 1).

**Table 1:** Study characteristics of included studies.

First author (year)	Country	Study design	Sample size, statinusers/non-users	Age	Male (%)	Diagnostic criteria	Inclusion/exclusioncriteria	Follow-up duration	Exposure definition (statin type, timing, duration)	Type of statin	Ethnicity (%)	Site of onset (%)	BaselineALSFRS-R	ALS diseaseduration at baseline	Othercomorbidities	Use of disease-modifying therapies	Adjusted covariates	Summary of findings	Quality assessment
Qureshi (2008)	USA	Pooled analysis of three RCTs + one observational cohort	596, 36/560	Mean ± SD: 56.0 ± 12.2	64%	NM	Volunteers with ALS/receiving topiramate (*n* = 199)	Up to 1 year	Any statin use at baseline	NM	Caucasian (92%)	Limb (80.5%)	31.2 ± 5.3	NM	NM	Riluzole (entered as covariate in analysis; no effect on survival or function)	Age, weight, time from onset to diagnosis, baseline ALSFRS score, vital capacity, gender, baseline riluzole use, past medical conditions, and clinical study	No significant survival association with statin use	7
Vaage (2025)	Norway	Population-based cohort study	524, 191/322	Mean ± SD: 67.0 ± 8.1	57%	ICD-10 criteria	At least two G12.2 registrations/registration before January 1st, 2009, initiation of a statin after an ALS diagnosis, prior riluzole use, initiation of a statin after an ALS diagnosis	Mean: 2.0 years, max 11.8 years	Statin use at or within 1 year before diagnosis, at least two statin dispensations, and a cumulative dispensation of no less than 60 defined daily doses	NM	NM	NM	NM	1.9–2.1 years	NM	Riluzole in 74.4% of statin users and 72.4% of non-statin users	Sex, age at diagnosis, birth year, health screening, BMI, smoking, total cholesterol levels, and riluzole use	No significant survival association with statin use	8
Weisskopf (2021)	Israel	Population-based cohort study	948, 435/513	Mean ± SD: 64.1 ± 12.6	59.4%	ICD-9 criteria	Age 25 or higher, 9th Revision (ICD-9) [code of 335.20] AND/OR riluzole use/first indication of ALS before 2004 or less than 4 years of membership in CHS immediately prior to the ALS diagnosis	Median: statin users: 2.2 years, non-users: 3.2 years	Statin use ≤ 3 years before diagnosis	simvastatin, lovastatin, pravastatin, fluvastatin, atorvastatin, cerivastatin, and rosuvastatin	Arab (10.5%), Ashkenazi (28.1%), Sephardi (31.2%), Other (30.2%)	NM	NM	NM	Diabetes (23.7%), chronic renal failure (9.8%), and cardiovas- -cular disease (67%)	Riluzole use, nonstatin lipid-lowering medications ω-3 triglycerides and ezetimibe	Age, sex, socioeconomic status, smoking status, ethnicity, immigration status, ezetimibe, and nonstatin lipid-lowering medications, diagnosis of diabetes, chronic renal failure, cardiovascular disease, and residential district	Possible reduced mortality with earlier/longer statin use.	7
Schumacher (2020)	Ger- -many	Population-based cohort study	501, 65/436	Mean ± SD: 65.2 ± 10.9	58.5%	El Escorial Criteria	Diagnosis of ALS based on El escorial criteria/insufficient data of ALSFRS-R	Median (IQR): 14.3 (7.4–22.3) years	Self-reported use of Statin	simvastatin (*n* = 54), atorvastatin (*n* = 6), pravastatin (*n* = 3), fluvastatin (*n* = 2)	NM	Spinal (66.9%), bulbar (31.9%), unknown (1.2%)	Median (IQR): 39.0 (34.0–43.0)	Median (IQR): 6.0 (3.0–10.0)	Diabetes (11.8%)	NM	Age, gender, BMI, site of onset, ALSFRS-R at inclusion, and progression rate	No significant survival association with statin use.	7
Seelen (2014)	Nether- -lands	Population-based case–control study	722, 90/632	Median (IQR): 62.8 (56.7–69.6)	60.2%	Revised El Escorial Criteria	Patients who had a first, second or third degree family member with ALS, defined as familial ALS.	NM	Lipid modifying agents	ATC code C10	NM	Bulbar (32.8%)	NM	NM	Cardiovascular diseases (48.8%), neurode- -generative diseases (0.8%), psychiatric disorders (2.2%), cancer (7.6%), infectious diseases (10.4), autoimmune diseases (2.1%), trauma (11.2%), surgery (38.5%)	NM	Gender, age at onset, site of onset	No significant survival association with statin use.	6
Drory (2008)	Israel	Retrospective cohort study	459, 72/387	Mean ± SD: 58.8 ± 13 years		El Escorial Criteria	Patients with probable or definite ALS according to El Escorial criteria/patients aged less than 45 years	Up to 10 years	Any statin use at disease onset	Lipophilic statins; simvastatin (*n* = 52), pravastatin (*n* = 13), atorvastatin (*n* = 6), and lovastatin (*n* = 1)	NM	Bulbar (23.6%−27.6%)	NM	NM	NM	Riluzole (88.9% on riluzole; *n* = 64)	Age, gender, disease form at onset	No significant survival association with statin use.	5

NM, not mentioned.

The quality of the included studies was assessed using the Newcastle-Ottawa Scale for cohort studies. Most studies were rated as having a moderate to high quality, with adequate control for confounders. However, some studies had limitations due to the observational nature of the data, including potential bias in statin exposure assessment and the lack of randomization (Table 1).

**Figure 2:**
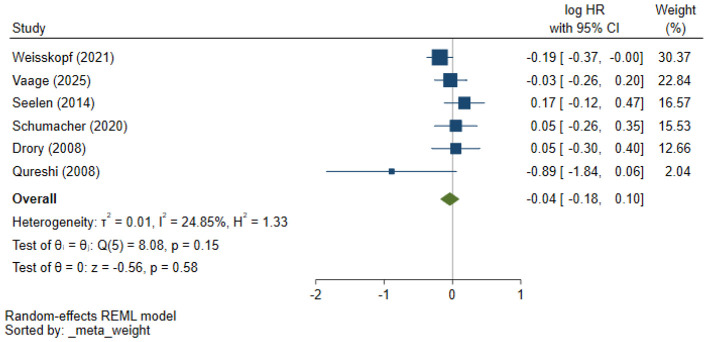
Forest plot of included studies indicating a pooled effect of statin use on ALS survival.

The meta-analysis of the six studies revealed a pooled effect of statin use on ALS survival. The Log (HR) for statin use compared to non-use was calculated using a random-effects model. Statin use was associated with a non-significant trend toward improved survival [Log (HR) = −0.04, 95% CI: −0.18 to 0.10]. There was moderate heterogeneity among the studies (*I*^2^ = 24.85%), suggesting variability in the results, likely due to differences in study designs, patient characteristics, and follow-up durations (Figure 2).

Exploratory meta-regression (hypothesis-generating only) was performed to assess whether study-level mean age or sex explained heterogeneity; however, results are underpowered and should be interpreted cautiously. The analysis demonstrated that neither age (β = 0.015, 95% CI −0.117 to 0.146; *p* = 0.826), nor sex (β = −0.021, 95% CI −0.166 to 0.123; *p* = 0.771) were not significant predictors of variation in the effect sizes (log HR). The Wald χ^2^ statistic (χ^2^ = 1.03, *p* = 0.597) showed that the regression model as a whole did not significantly account for between-study variability, suggesting that these demographic covariates did not explain heterogeneity in the pooled results.

Sensitivity analyses were performed to evaluate the influence of individual studies on the overall results. The exclusion of each study did not substantially change the pooled effect, indicating that the overall pooled effect remained stable and consistent (Supplementary Figure S1).

The funnel plot displays the distribution of the studies included in the meta-analysis. The plot appears largely symmetrical, suggesting a low likelihood of publication bias. Only one study is more widely scattered at the bottom, whereas larger studies cluster toward the top, as expected in a well-conducted meta-analysis (Supplementary Figure S2).

Using the GRADE approach, the certainty of evidence for the pooled effect of statin use on ALS survival was judged to be very low. Because all six included studies were observational, the starting level of certainty was low, and we downgraded further for risk of bias due to the non-randomized nature of the evidence and the high likelihood of residual confounding (e.g., confounding by indication and differences in comorbidities and co-interventions), as well as potential exposure misclassification given heterogeneity in how statin exposure was defined across studies. We also downgraded for inconsistency, because there was moderate between-study heterogeneity (*I*^2^ = 24.85%), consistent with variability in designs, populations, and follow-up durations. Finally, we downgraded for imprecision, because the pooled estimate was close to null, limiting confidence in the direction and magnitude of effect. Although the funnel plot appeared largely symmetrical, with only six studies the ability to detect publication bias is limited; therefore, we did not upgrade certainty.

## 4 Discussion

ALS remains a devastating disease with poorly understood mechanisms and limited therapies that meaningfully slow progression. This has increased interest in repurposing widely used medications, including statins, which have cholesterol-independent (pleiotropic) anti-inflammatory and potentially neuroprotective effects. However, prior studies on statins and ALS survival have been inconsistent, reporting benefit, harm, or no association (19, 20). Our results, which indicate that statins have no significant positive or negative effects on ALS survival, add to the current discussion and draw attention to a number of key gaps in the research.

Our comprehensive literature review on statins and ALS survival has emphasized these conflicting results, reflecting a variety of methodological differences and the complex nature of ALS itself. While several studies have suggested a protective effect of statins, others have reported no effect, and some even indicated that statins might worsen ALS outcomes. For instance, a study by Drory et al. (7) found no difference in survival between patients with ALS treated with statins and those not treated. Similarly, Schumacher et al. (21) reported that statin use did not significantly affect overall survival in a cohort of 501 patients with ALS. On the other hand, some studies have raised the possibility that statins might confer a survival benefit when administered early in the disease course (11, 22).

The observational nature of the majority of studies may be one of the main factors contributing to the contradictory results. Cohort studies can provide insightful information, but they are prone to confounding variables (23) that could mask the real connection between ALS survival and statin use. In addition, adjustment strategies differed across studies, and important confounders (e.g., comorbid cardiovascular disease/diabetes risk profiles, healthcare utilization, ALS phenotype and severity measures, and concomitant therapies) were not consistently measured or modeled. Therefore, even when adjusted ratios were used, residual confounding (including confounding by indication and reverse causality related to statin discontinuation) may remain and could bias estimates toward or away from the null. Therefore, the results should be interpreted with caution. For instance, these studies may not adequately account for the differences between individuals who are prescribed statins and those who are not. Moreover, statin use is closely linked to underlying cardiometabolic conditions (e.g., dyslipidemia, diabetes mellitus, and cardiovascular risk profiles). This creates a risk of confounding by indication in observational studies, because factors that prompt statin prescribing and their associated comorbidity burden, may also be associated with ALS diagnosis timing, baseline health status, and measured outcomes, independent of any causal effect of statins. Therefore, the prognosis of ALS is also impacted by the underlying medical condition that requires the statin use, which still makes a definite conclusion more difficult.

Furthermore, the heterogeneous nature of ALS adds another layer of complexity. ALS is a spectrum of disorders rather than a single disease, varying in terms of site of onset (bulbar vs. limb), disease progression, and clinical symptoms. Differences in some key parameters in patients with ALS, along with variations in disease duration and severity at the time of statin initiation, could explain the double-edged effect of statins in different studies. Additionally, using riluzole or edaravone, the two FDA-approved drug for ALS might simultaneously influence the interpretation of results, as they have been shown to slightly prolong survival (3) but may interact with the effects of statins in ways that are not entirely known.

Our meta-analysis found no significant findings regarding the beneficial or harmful effects of statins on ALS survival. This result highlights several key gaps and raises questions on the topic.

Several previous systematic reviews/meta-analyses have primarily examined statin exposure in relation to ALS susceptibility/incidence/risk, and overall, they do not support a clear association. In previous systematic reviews, such as those by Zheng et al. (24), the available evidence on the relationship between statins and ALS incidence was inconclusive, with pooled analyses yielding non-significant associations. Similarly, Chang et al. (25) synthesized three case-control and one cohort study and found no statistically significant difference in statin exposure between ALS and control groups (OR 0.75; 95% CI 0.53–1.08). A more recent meta-analysis by Nabizadeh et al. (26), which incorporated a larger body of observational evidence (eight studies; 13,890 ALS cases), also reported a null association (RR 0.98; 95% CI 0.80–1.20) but noted substantial between-study heterogeneity (*I*^2^ 72.71%). In addition, a recent large active-comparator, new-user registry study from East Asia compared statin initiators with ezetimibe or fibrate initiators and reported a lower hazard of ALS onset among statin users (27).

Collectively, these incidence-focused syntheses suggest that any effect of statins on ALS risk is uncertain and likely small, with heterogeneity driven by differences in exposure definitions, study design, and confounding control. In contrast, the present review is intentionally focused on a different endpoint, survival after ALS diagnosis, using time-to-event outcomes including recent population-based survival cohorts.

Emerging studies have associated lipid metabolism with ALS risk and progression, though findings vary depending on disease stage and lipid profile measures. Premorbid lipid levels and lipid-related genetic instruments have been associated with ALS susceptibility, with several large-scale analyses suggesting that higher LDL may be linked to increased ALS risk (14). In contrast, within ALS cohorts, lipid measures may relate differently to prognosis/survival: some studies have reported longer survival in patients with higher triglycerides and/or total cholesterol, whereas others have found null associations or observed that higher HDL may correlate with poorer prognosis, highlighting heterogeneity and potential confounding by nutritional status, weight loss, and disease severity at measurement (15). Another key point is that, according to some research, higher cholesterol levels may be protective against the progression of the disease (13), and cholesterol metabolism has been linked to the pathophysiology of ALS (28). Theoretically, statins could potentially interfere with the protective effects of cholesterol by lowering cholesterol levels. On the other hand, other studies have suggested that elevated cholesterol levels may contribute to the pathogenesis of ALS by promoting neuroinflammation and oxidative stress (29). These distinctions are clinically relevant because the effects of statins in ALS, if any, could reflect a balance between (i) cholesterol lowering (which might be unfavorable in some patients if it tracks declining metabolic reserve) and (ii) cholesterol-independent pleiotropic actions (e.g., immunomodulatory and endothelial effects) (30). Accordingly, evidence on lipid levels and ALS incidence should be interpreted separately from evidence on statin exposure and post-diagnosis survival, and future studies should report both lipid trajectories and time-updated statin exposure to clarify causal direction.

One of the main drawbacks of the existing studies on ALS survival and statin use is the variety in study designs and methodologies. While only one study pooled the data from three clinical trials and one observational study (9), the majority of research on statins and ALS survival is observational (7–9, 11, 22). RCTs are the highest standard for determining causality (31). However, they are often difficult to conduct in ALS due to the rare nature of the disease and the ethical issues around withholding potential treatments. Observational studies have tried to close this gap in the absence of RCTs, but they are subject to bias and confounding factors. Many observational studies, for example, fail to appropriately account for factors like the existence of other comorbidities, the dosage of statins used, or the time of statin beginning. These elements have the potential to significantly affect the outcomes and make it challenging to reach firm conclusions. Another remarkable gap is the lack of consensus on the type and dosage of statins used in these studies. Since statins are a heterogeneous class of medications, they vary in potency, pharmacokinetics, and side effect profiles. Statins differ in physicochemical properties that influence tissue distribution. In general, lipophilic statins (e.g., simvastatin, atorvastatin, lovastatin, fluvastatin, pitavastatin) more readily diffuse across cell membranes, whereas hydrophilic statins (notably pravastatin and rosuvastatin) are more hepatoselective and depend more on transporter-mediated uptake, which may limit extrahepatic exposure. These differences are relevant because lipophilicity has been linked to greater potential for CNS exposure, which could theoretically enhance statin “pleiotropic” actions in the nervous system (e.g., modulation of neuroinflammation and endothelial function), but may also increase the likelihood of CNS-related adverse effects in sensitive individuals (32). Beyond lipid-lowering effects, statins modulate signaling pathways like Rho/Rac through the reduction of isoprenoid intermediates, potentially impacting neuroinflammation and oxidative stress (33). Experimental work has also compared statins for putative neuroprotective profiles, including differences related to lipophilicity and estimated blood–brain barrier penetration. Taken together, lipophilicity and CNS exposure represent plausible effect modifiers and a potential source of heterogeneity; future prognostic studies should report survival estimates stratified by statin class (lipophilic vs. hydrophilic) and incorporate time-varying exposure to reduce bias. In addition, the dosage of statins is also important; while higher dosages may have a greater influence on cholesterol levels, they may also raise the likelihood of undesirable side effects, including myopathy, which could have a detrimental effect on patients with ALS (34). Additionally, statins differ substantially in potency, and pooling them as a single exposure may mask subgroup effects. Weisskopf et al. (11) reported that use of only lower-potency statins was associated with longer survival after ALS diagnosis, whereas the association with higher-potency statins was null. Because potency-stratified survival estimates were not consistently available across other included studies, we could not meta-analyze potency subgroups. Nevertheless, potency remains a plausible contributor to between-study heterogeneity and should be prioritized in future prognostic analyses. It is challenging to determine whether some statin types are more advantageous or detrimental than others because many studies do not differentiate between various statins or their dosages.

An important concern in observational studies is that early ALS-related muscle symptoms may be misinterpreted as statin-associated myopathy, prompting discontinuation of statins around the time of symptom onset or diagnosis. This “statin discontinuation” phenomenon can introduce reverse causality and selection bias, because patients who remain classified as statin users may differ systematically from those who discontinue (e.g., healthier survivors, different healthcare utilization, or different disease trajectories). As most included studies relied on baseline or pre-diagnostic exposure definitions and did not model statin use as a time-varying exposure with appropriate lag periods, residual bias from discontinuation cannot be excluded and may contribute to heterogeneous findings.

Another crucial factor that hasn't been adequately covered in the literature is the time of statin initiation. According to Weisskopf et al.'s (11) study, using statins early on may prolong survival in patients with ALS. This effect might be due to reducing neuroinflammation and oxidative stress. However, when statins are started later in the course of ALS, it is less evident how they will affect the disease's progression over the long-term follow-ups. Due to a lack of data on the initiation of statins among the included studies and the dispersion of data among others, we could not assess its impact on survival. However, regardless of when statin use occurred, our meta-analysis did not find any evidence that it significantly affected survival. This might be because several of the included studies showed advanced disease, which might have made statin treatment less helpful in changing the course of the disease. In order to ascertain whether there is a crucial window of opportunity for intervention, future research should concentrate on the possible advantages of starting statins early in ALS.

Furthermore, the use of other concomitant medications, particularly riluzole, could distort the results of studies assessing the effects of statins on ALS survival. Riluzole has been shown to modestly extend survival (3), but it is unknown how it interacts with statins. However, the lack of consistent data on the interaction between statins and riluzole makes it difficult to determine whether statins exert an independent effect on survival or if their effects are modified by other treatments.

It should be noted that, this review was restricted to English-language, peer-reviewed full-text articles, which may introduce language or publication bias. Although this approach improves reliability of survival data extraction and risk-of-bias assessment, it may exclude relevant evidence published in other languages or available only as abstracts.

The most likely interpretation of the pooled estimate is that statin use does not have a clinically meaningful effect on survival in ALS at the population level. Nonetheless, several factors could still contribute to variability across observational studies and may obscure subgroup-specific associations. We discussed the possible reasons as follows:

Theoretically, statins are known to have anti-inflammatory and neuroprotective effects that could benefit patients with ALS. They have been shown to reduce the production of pro-inflammatory cytokines and suppress the activation of microglia, which played a key role in neuroinflammation in ALS (35). Furthermore, considering the antioxidant effects of statins, they could mitigate oxidative stress, which could protect motor neurons from degeneration in ALS (36). However, the neuroprotective effects of statins in ALS are not well-established, and it is possible that these effects are not strong enough to significantly alter survival, particularly in patients with advanced disease. Additionally, the benefit of statins in ALS (if any) may be dependent on other factors, such as the type of statins used, and the presence of comorbid conditions, which were not consistently accounted for in the studies included in our meta-analysis.

On the other hand, muscle toxicity, which can range from mild myalgia to more severe conditions such as myopathy and rhabdomyolysis is among the known side effects of statins. Given that ALS is a motor neuron disease that affects the motor neurons controlling voluntary muscle movement, statin-induced muscle damage could exacerbate the disease course and negatively impact quality of life. Several previous studies have shown that ALS can statins may worsened muscle strength and increased fall risks in older adults (37, 38). The muscle-related side effects of statins could explain why some studies found no survival benefit or even harm associated with statin use. The presence of muscle weakness and fatigue could lead to a reduction in physical function, potentially accelerating the progression of ALS.

Finally, a lack of survival difference does not exclude clinically meaningful effects on functional decline, tolerability, discontinuation, or quality of life; future studies should report functional endpoints (e.g., ALSFRS-R trajectories), respiratory milestones, and patient-reported outcomes alongside mortality.

## 5 Conclusion

In this meta-analysis, the pooled estimate did not demonstrate a statistically significant association between statin use and survival among patients with ALS. The most likely interpretation of the pooled estimate is that statin use does not have a clinically meaningful effect on survival in ALS at the population level. However, the interpretation should remain cautious because the included studies differed in statin exposure definitions (including type/potency, duration, and timing), patient characteristics, and covariate adjustment, and most evidence was observational. As a result, substantial residual confounding is likely, and a modest benefit or harm in specific subgroups cannot be excluded. Current evidence therefore does not support initiating statins solely to modify ALS survival; statins should be prescribed based on standard cardiovascular indications with individualized clinical judgment. Future studies with clearer time-varying exposure ascertainment and more consistent control of key confounders are needed. Future studies should address the methodological gaps identified in this analysis, including the use of randomized controlled trials, the standardization of statin type and dosage, and the consideration of the timing of statin initiation. Additionally, studies should better account for the potential bias due to interactions between statins and other treatments, such as riluzole, and investigate the underlying mechanisms that could explain the lack of a clear effect on ALS survival.

## Data Availability

The original contributions presented in the study are included in the article/Supplementary material, further inquiries can be directed to the corresponding author.
