# Supplementary material for: The effect of statins on the survival of patients with amyotrophic lateral sclerosis: a meta-analysis
Source: Front Neurol. 2026 Apr 1;17:1753992. doi: 10.3389/fneur.2026.1753992 (PMC13078970; doi:10.3389/fneur.2026.1753992)
Supplement: Supplementary file 2 [file Table_2.docx]

Supplementary Material

# Supplementary tables

| **Table S1- Main and alternative keywords used in search** | | |
| --- | --- | --- |
| Amyotrophic Lateral Sclerosis | **Statin** | **Survival** |
| Amyotrophic Lateral Sclerosis  ALS  Gehrig's Disease  Gehrig Disease  Gehrigs Disease  Lou Gehrig Disease  Motor Neuron Disease  Lou Gehrig's Disease  Lou-Gehrigs Disease  Charcot Disease  Amyotrophic Lateral Sclerosis With Dementia  Dementia With Amyotrophic Lateral Sclerosis  Guam Disease  Amyotrophic Lateral Sclerosis-Parkinsonism-Dementia Complex 1  Amyotrophic Lateral Sclerosis Parkinsonism Dementia Complex 1  Amyotrophic Lateral Sclerosis, Parkinsonism-Dementia Complex of Guam  Amyotrophic Lateral Sclerosis, Parkinsonism Dementia Complex of Guam  Guam Form of Amyotrophic Lateral Sclerosis | Hydroxymethylglutaryl CoA Reductase Inhibitor*  HMG-CoA Reductase Inhibitor*  HMG CoA Reductase Inhibitor*  Hydroxymethylglutaryl-Coenzyme A Inhibitors  Hydroxymethylglutaryl-CoA Inhibitors  Hydroxymethylglutaryl-CoA Reductase Inhibitor  Statin*  HMG-CoA Statin*  Lipid-lowering drugs  Simvastatin  Atorvastatin  Lovastatin  Fluvastatin  Pravastatin  Rosuvastatin  Pitavastatin | Survival  Survival Rate*  Cumulative Survival Rate*  Mean Survival Time*  Mortalit*  Mortality Rate*  Death Rate*  Differential Mortalit*  Excess Mortalit*  Mortality Determinant*  Case Fatality Rate*  CFR  Mortality Decline*  Age-Specific Death Rate*  Age Specific Death Rate*  Crude Death Rate*  Crude Mortality Rate* |

| Table S2- Detailed search strategy in each database | | |
| --- | --- | --- |
| Database | **Search strategy** | **Results** |
| PubMed | #1 ((((((((((((((Amyotrophic Lateral Sclerosis[Title/Abstract]) OR (ALS[Title/Abstract])) OR (Gehrig's Disease[Title/Abstract])) OR (Gehrig Disease[Title/Abstract])) OR (Gehrigs Disease[Title/Abstract])) OR (Lou Gehrig Disease[Title/Abstract])) OR (Motor Neuron Disease[Title/Abstract])) OR (Lou Gehrig's Disease[Title/Abstract])) OR (Lou-Gehrigs Disease[Title/Abstract])) OR (Charcot Disease[Title/Abstract])) OR (Amyotrophic Lateral Sclerosis With Dementia[Title/Abstract])) OR (Dementia With Amyotrophic Lateral Sclerosis[Title/Abstract])) OR (Guam Disease[Title/Abstract])) OR (Amyotrophic Lateral Sclerosis-Parkinsonism-Dementia Complex 1[Title/Abstract])) OR (Amyotrophic Lateral Sclerosis Parkinsonism Dementia Complex 1[Title/Abstract]) | [52,868](https://pubmed.ncbi.nlm.nih.gov/?term=%28%28%28%28%28%28%28%28%28%28%28%28%28%28Amyotrophic+Lateral+Sclerosis%5BTitle%2FAbstract%5D%29+OR+%28ALS%5BTitle%2FAbstract%5D%29%29+OR+%28Gehrig%27s+Disease%5BTitle%2FAbstract%5D%29%29+OR+%28Gehrig+Disease%5BTitle%2FAbstract%5D%29%29+OR+%28Gehrigs+Disease%5BTitle%2FAbstract%5D%29%29+OR+%28Lou+Gehrig+Disease%5BTitle%2FAbstract%5D%29%29+OR+%28Motor+Neuron+Disease%5BTitle%2FAbstract%5D%29%29+OR+%28Lou+Gehrig%27s+Disease%5BTitle%2FAbstract%5D%29%29+OR+%28Lou-Gehrigs+Disease%5BTitle%2FAbstract%5D%29%29+OR+%28Charcot+Disease%5BTitle%2FAbstract%5D%29%29+OR+%28Amyotrophic+Lateral+Sclerosis+With+Dementia%5BTitle%2FAbstract%5D%29%29+OR+%28Dementia+With+Amyotrophic+Lateral+Sclerosis%5BTitle%2FAbstract%5D%29%29+OR+%28Guam+Disease%5BTitle%2FAbstract%5D%29%29+OR+%28Amyotrophic+Lateral+Sclerosis-Parkinsonism-Dementia+Complex+1%5BTitle%2FAbstract%5D%29%29+OR+%28Amyotrophic+Lateral+Sclerosis+Parkinsonism+Dementia+Complex+1%5BTitle%2FAbstract%5D%29&sort=relevance) |
|  | #2 (((((((((((((((Hydroxymethylglutaryl CoA Reductase Inhibitor*[Title/Abstract]) OR (HMG-CoA Reductase Inhibitor*[Title/Abstract])) OR (HMG CoA Reductase Inhibitor*[Title/Abstract])) OR (Hydroxymethylglutaryl-Coenzyme A Inhibitors[Title/Abstract])) OR (Hydroxymethylglutaryl-CoA Inhibitors[Title/Abstract])) OR (Hydroxymethylglutaryl-CoA Reductase Inhibitor[Title/Abstract])) OR (Statin*[Title/Abstract])) OR (HMG-CoA Statin*[Title/Abstract])) OR (Lipid-lowering drugs[Title/Abstract])) OR (Simvastatin[Title/Abstract])) OR (Atorvastatin[Title/Abstract])) OR (Lovastatin[Title/Abstract])) OR (Fluvastatin[Title/Abstract])) OR (Pravastatin[Title/Abstract])) OR (Rosuvastatin[Title/Abstract])) OR (Pitavastatin[Title/Abstract]) | [79,454](https://pubmed.ncbi.nlm.nih.gov/?term=%28%28%28%28%28%28%28%28%28%28%28%28%28%28%28Hydroxymethylglutaryl+CoA+Reductase+Inhibitor%2A%5BTitle%2FAbstract%5D%29+OR+%28HMG-CoA+Reductase+Inhibitor%2A%5BTitle%2FAbstract%5D%29%29+OR+%28HMG+CoA+Reductase+Inhibitor%2A%5BTitle%2FAbstract%5D%29%29+OR+%28Hydroxymethylglutaryl-Coenzyme+A+Inhibitors%5BTitle%2FAbstract%5D%29%29+OR+%28Hydroxymethylglutaryl-CoA+Inhibitors%5BTitle%2FAbstract%5D%29%29+OR+%28Hydroxymethylglutaryl-CoA+Reductase+Inhibitor%5BTitle%2FAbstract%5D%29%29+OR+%28Statin%2A%5BTitle%2FAbstract%5D%29%29+OR+%28HMG-CoA+Statin%2A%5BTitle%2FAbstract%5D%29%29+OR+%28Lipid-lowering+drugs%5BTitle%2FAbstract%5D%29%29+OR+%28Simvastatin%5BTitle%2FAbstract%5D%29%29+OR+%28Atorvastatin%5BTitle%2FAbstract%5D%29%29+OR+%28Lovastatin%5BTitle%2FAbstract%5D%29%29+OR+%28Fluvastatin%5BTitle%2FAbstract%5D%29%29+OR+%28Pravastatin%5BTitle%2FAbstract%5D%29%29+OR+%28Rosuvastatin%5BTitle%2FAbstract%5D%29%29+OR+%28Pitavastatin%5BTitle%2FAbstract%5D%29&sort=) |
|  | #3 ((((((((((((((((Survival[Title/Abstract]) OR (Survival Rate*[Title/Abstract])) OR (Cumulative Survival Rate*[Title/Abstract])) OR (Mean Survival Time*[Title/Abstract])) OR (Mortalit*[Title/Abstract])) OR (Mortality Rate*[Title/Abstract])) OR (Death Rate*[Title/Abstract])) OR (Differential Mortalit*[Title/Abstract])) OR (Excess Mortalit*[Title/Abstract])) OR (Mortality Determinant*[Title/Abstract])) OR (Case Fatality Rate*[Title/Abstract])) OR (CFR[Title/Abstract])) OR (Mortality Decline*[Title/Abstract])) OR (Age-Specific Death Rate*[Title/Abstract])) OR (Age Specific Death Rate*[Title/Abstract])) OR (Crude Death Rate*[Title/Abstract])) OR (Crude Mortality Rate*[Title/Abstract]) | [2,327,655](https://pubmed.ncbi.nlm.nih.gov/?term=%28%28%28%28%28%28%28%28%28%28%28%28%28%28%28%28Survival%5BTitle%2FAbstract%5D%29+OR+%28Survival+Rate%2A%5BTitle%2FAbstract%5D%29%29+OR+%28Cumulative+Survival+Rate%2A%5BTitle%2FAbstract%5D%29%29+OR+%28Mean+Survival+Time%2A%5BTitle%2FAbstract%5D%29%29+OR+%28Mortalit%2A%5BTitle%2FAbstract%5D%29%29+OR+%28Mortality+Rate%2A%5BTitle%2FAbstract%5D%29%29+OR+%28Death+Rate%2A%5BTitle%2FAbstract%5D%29%29+OR+%28Differential+Mortalit%2A%5BTitle%2FAbstract%5D%29%29+OR+%28Excess+Mortalit%2A%5BTitle%2FAbstract%5D%29%29+OR+%28Mortality+Determinant%2A%5BTitle%2FAbstract%5D%29%29+OR+%28Case+Fatality+Rate%2A%5BTitle%2FAbstract%5D%29%29+OR+%28CFR%5BTitle%2FAbstract%5D%29%29+OR+%28Mortality+Decline%2A%5BTitle%2FAbstract%5D%29%29+OR+%28Age-Specific+Death+Rate%2A%5BTitle%2FAbstract%5D%29%29+OR+%28Age+Specific+Death+Rate%2A%5BTitle%2FAbstract%5D%29%29+OR+%28Crude+Death+Rate%2A%5BTitle%2FAbstract%5D%29%29+OR+%28Crude+Mortality+Rate%2A%5BTitle%2FAbstract%5D%29&sort=) |
|  | #1 AND #2 AND #3 | 20 |
| Scopus | #1 ( TITLE-ABS-KEY ( Amyotrophic Lateral Sclerosis ) OR TITLE-ABS-KEY ( ALS ) OR TITLE-ABS-KEY ( Gehrig Disease ) OR TITLE-ABS-KEY ( Lou Gehrig Disease ) OR TITLE-ABS-KEY ( Motor Neuron Disease ) OR TITLE-ABS-KEY ( Charcot Disease ) OR TITLE-ABS-KEY ( Guam Disease ) OR TITLE-ABS-KEY ( Amyotrophic Lateral Sclerosis Parkinsonism Dementia Complex 1 ) ) | 247,659 results |
|  | #2 ( TITLE-ABS-KEY ( Hydroxymethylglutaryl CoA Reductase Inhibitor* ) OR TITLE-ABS-KEY ( HMG-CoA Reductase Inhibitor* ) OR TITLE-ABS-KEY ( HMG CoA Reductase Inhibitor* ) OR TITLE-ABS-KEY ( Hydroxymethylglutaryl-Coenzyme A Inhibitors ) OR TITLE-ABS-KEY ( Hydroxymethylglutaryl-CoA Inhibitors ) OR TITLE-ABS-KEY ( Hydroxymethylglutaryl-CoA Reductase Inhibitor ) OR TITLE-ABS-KEY ( Statin* ) OR TITLE-ABS-KEY ( HMG-CoA Statin* ) OR TITLE-ABS-KEY ( Lipid-lowering drugs ) OR TITLE-ABS-KEY ( Simvastatin ) OR TITLE-ABS-KEY ( Atorvastatin ) OR TITLE-ABS-KEY ( Lovastatin ) OR TITLE-ABS-KEY ( Fluvastatin ) OR TITLE-ABS-KEY ( Pravastatin ) OR TITLE-ABS-KEY ( Rosuvastatin ) OR TITLE-ABS-KEY ( Pitavastatin ) ) | 225,416 results |
|  | #3 ( TITLE-ABS-KEY ( Survival ) OR TITLE-ABS-KEY ( Survival Rate* ) OR TITLE-ABS-KEY ( Cumulative Survival Rate* ) OR TITLE-ABS-KEY ( Mean Survival Time* ) OR TITLE-ABS-KEY ( Mortalit* ) OR TITLE-ABS-KEY ( Mortality Rate* ) OR TITLE-ABS-KEY ( Death Rate* ) OR TITLE-ABS-KEY ( Mortality Determinant* ) OR TITLE-ABS-KEY ( Case Fatality Rate* ) OR TITLE-ABS-KEY ( Mortality Decline* ) OR TITLE-ABS-KEY ( Age Specific Death Rate* ) OR TITLE-ABS-KEY ( Crude Death Rate* ) OR TITLE-ABS-KEY ( Crude Mortality Rate* ) ) | 4,065,067 results |
|  | #1 AND #2 AND #3 | 132 results |
| Web of Science | #1  Amyotrophic Lateral Sclerosis (Topic) or ALS (Topic) or Gehrig's Disease (Topic) or Gehrig Disease (Topic) or Gehrigs Disease (Topic) or Lou Gehrig Disease (Topic) or Motor Neuron Disease (Topic) or Lou Gehrig's Disease (Topic) or Lou-Gehrigs Disease (Topic) or Charcot Disease (Topic) or Amyotrophic Lateral Sclerosis With Dementia (Topic) or Dementia With Amyotrophic Lateral Sclerosis (Topic) or Guam Disease (Topic) or Amyotrophic Lateral Sclerosis-Parkinsonism-Dementia Complex 1 (Topic) or Amyotrophic Lateral Sclerosis Parkinsonism Dementia Complex 1 (Topic) | [108,252](https://www.webofscience.com/wos/woscc/summary/a2607c12-9344-4366-b2d0-47ed53e60f17-0174e5cd91/relevance/1) |
|  | #2  Hydroxymethylglutaryl CoA Reductase Inhibitor* (Topic) or HMG-CoA Reductase Inhibitor* (Topic) or HMG CoA Reductase Inhibitor* (Topic) or Hydroxymethylglutaryl-Coenzyme A Inhibitors (Topic) or Hydroxymethylglutaryl-CoA Inhibitors (Topic) or Hydroxymethylglutaryl-CoA Reductase Inhibitor (Topic) or Statin* (Topic) or HMG-CoA Statin* (Topic) or Lipid-lowering drugs (Topic) or Simvastatin (Topic) or Atorvastatin (Topic) or Lovastatin (Topic) or Fluvastatin (Topic) or Pravastatin (Topic) or Rosuvastatin (Topic) or Pitavastatin (Topic) | [120,256](https://www.webofscience.com/wos/woscc/summary/28e2e49e-b374-4e1c-978b-ce75265b7ae9-0174e5ed22/relevance/1) |
|  | #3  Survival (Topic) or Survival Rate* (Topic) or Cumulative Survival Rate* (Topic) or Mean Survival Time* (Topic) or Mortalit* (Topic) or Mortality Rate* (Topic) or Death Rate* (Topic) or Differential Mortalit* (Topic) or Excess Mortalit* (Topic) or Mortality Determinant* (Topic) or Case Fatality Rate* (Topic) or CFR (Topic) or Mortality Decline* (Topic) or Age-Specific Death Rate* (Topic) or Age Specific Death Rate* (Topic) or Crude Death Rate* (Topic) or Crude Mortality Rate* (Topic) | [3,297,022](https://www.webofscience.com/wos/woscc/summary/3a7ef650-015a-46cd-9d74-85b13dc53338-0174e605c7/relevance/1) |
|  | #1 AND #2 AND #3 | 41 |

# Supplementary figures


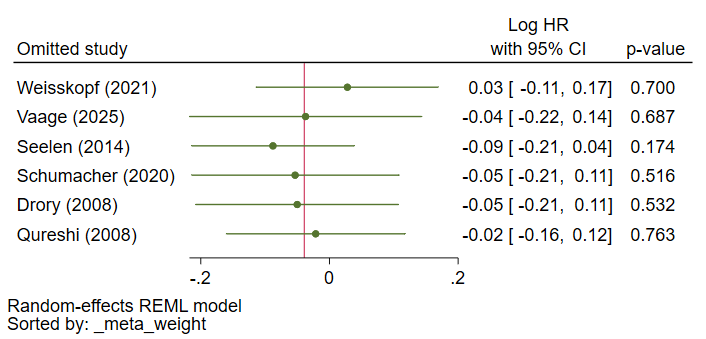


**Figure S1- Sensitivity analysis using leave one out test**

**
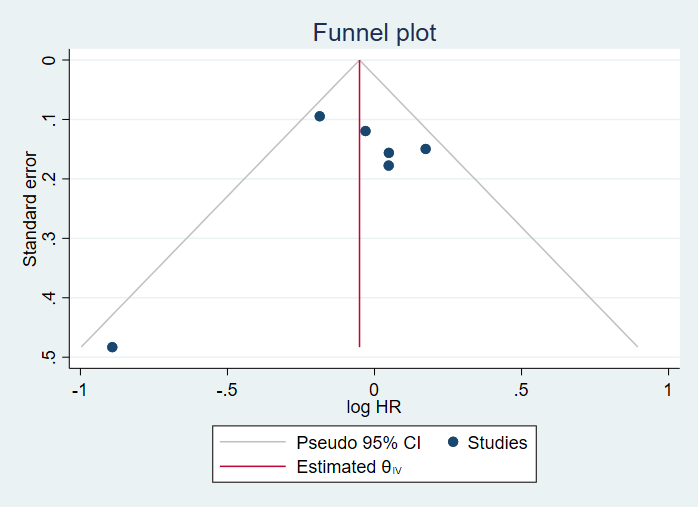
**

**Figure S2- Funnel plot of included studies**

Supplementary Material should be uploaded separately on submission. Please include any supplementary data, figures and/or tables.

**
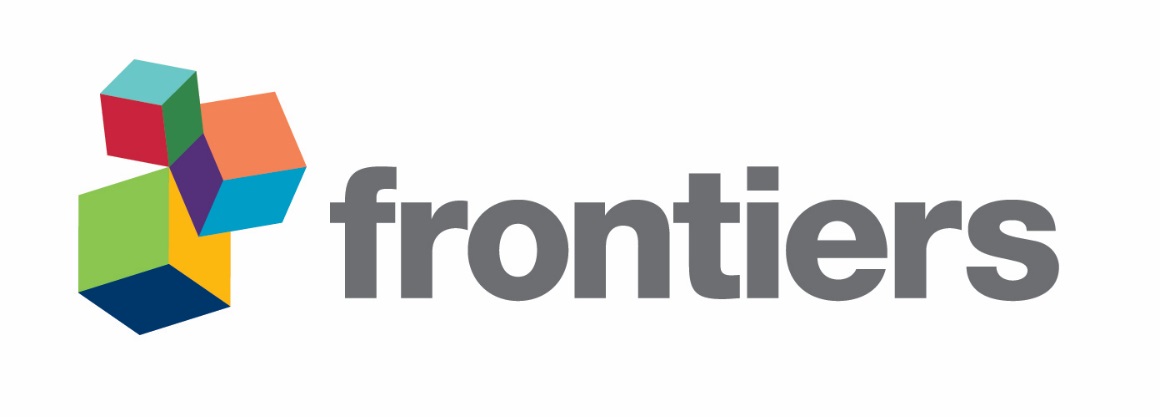
**
